# Clinical characteristics of food allergy post paediatric liver transplantation

**DOI:** 10.1186/2045-7022-3-S3-P114

**Published:** 2013-07-25

**Authors:** R De Bruyne, M Dullaers, S van Biervliet, S Vande Velde, P Gevaert, M van Winckel

**Affiliations:** 1 Department of Paediatric Gastroenterology, Hepatology and Nutrition, Ghent University Hospital, Ghent, Belgium; 2 Department of Pulmonary Medicine, Ghent University Hopsital, Ghent, Belgium; 3 Department of Otorhinolaryngology, Ghent University Hospital, Ghent, Belgium

## Background

Food allergy (FA) is a frequent finding after paediatric liver transplantation (LT). The pathogenesis remains incompletely understood. We here report the clinical characteristics of post transplant food allergy (PTFA) in children currently followed after LT in our centre.

## Methods

The study group consists of 49 LT patients transplanted at a median age of 22 months (3 weeks-16 years). Data were collected retrospectively from medical records and via a doctor’s questionnaire taken from the parents. The diagnosis of FA was based on convincing clinical symptoms followed by a clear response to elimination and provocation. Supervised open food challenge was performed when clinical history and symptomatology were not unequivocal and/or in case of IgE mediated acute symptoms. Skin prick testing (SPT) was performed with fresh products according to standard guidelines.

## Results

13 (26%) LT patients developed PTFA. None of the patients had symptoms prior to transplantation. 7/13 (54%) have multiple FA. Symptoms with acute onset (typical IgE mediated symptoms) are present in 6/13 (46%) compared to chronic symptoms with delayed/subacute onset in 7/13 (54%). In the latter, SPT and/or specific IgE were positive in 5/7 for clinically relevant antigens. 10/13 (77%) presented with gastro-intestinal symptoms (diarrhoea with faltering growth, bloody stools, vomiting), other symptoms were angioedema (4), urticaria (1), anaphylaxis (1) and severe atopic dermatitis (1). 3 patients suffer from treatment resistant cheilitis with oral mucosal lesions. One patient was diagnosed with eosinophilic oesophagitis (nodular aspect of oesophageal mucosa with longitudinal fissures on endoscopy, microscopy revealed > 20 eosinophils per high power field not responsive to treatment with proton pomp inhibitors). In at least 9/13 (69%) patients FA presented in the aftermath of a proven episode of gastroenteritis. The median follow-up since diagnosis of FA is 72 (33-188) months. In terms of prognosis, only 1 in 13 children has outgrown the FA, being able to reintroduce the food allergen in the diet.

## Conclusion

PTFA is an important clinical problem in children after LT. Patients present multiple, persistent FA mainly with gastrointestinal symptoms. Chronic oral lesions resistant to dietary intervention are seen in 25% of patients. IgE seems implicated in the immunopathology of PTFA in the majority of patients.

## Disclosure of interest

None declared.

